# Developments in Laminate Modification of Adhesively Bonded Composite Joints

**DOI:** 10.3390/ma16020568

**Published:** 2023-01-06

**Authors:** Farin Ramezani, Beatriz D. Simões, Ricardo J. C. Carbas, Eduardo A. S. Marques, Lucas F. M. da Silva

**Affiliations:** 1 Instituto de Ciência e Inovação em Engenharia Mecânica e Engenharia Industrial (INEGI), Rua Dr. Roberto Frias, 4200-465 Porto, Portugal; 2 Departamento de Engenharia Mecânica, Faculdade de Engenharia (FEUP), Universidade Do Porto, Rua Dr. Roberto Frias, 4200-465 Porto, Portugal

**Keywords:** composite materials, adhesively bonded joints, carbon fibre reinforced polymers

## Abstract

The use of carbon fibre reinforced polymer (CFRP) materials is increasing in many different industries, such as those operating in the aviation, marine, and automotive sectors. In these applications, composite parts are often joined with other composite or metallic parts, where adhesive bonding plays a key role. Unlike conventional joining methods, adhesive bonding does not add weight or require the drilling of holes, both of which are major sources of stress concentration. The performance of a composite joint is dependent on multiple factors and can be improved by modifying the adhesive layer or the composite layup of the adherend. Moreover, joint geometry, surface preparation, and the manufacturing methods used for production are also important factors. The present work reviews recent developments on the design and manufacture of adhesively bonded joints with composite substrates, with particular interest in adherend modification techniques. The effects of stacking sequence, use of thin-plies, composite metal laminates and its specific surface preparations, and the use of toughened surface layers in the composite adherends are described for adhesively bonded CFRP structures.

## 1. Introduction

Composites are high-performance and lightweight materials but ones that are hard to manufacture in large dimensions or in complex configurations [1]. From an industrial point of view, composite structures are often manufactured in multiple parts that will later be connected via different joining methods [2]. Joining of composites may take place during the manufacture of the original structure or during service, when repairing damage or replacing older components [3]. Multiple well-established joining methods are available for joining composites, such as riveting, fastening [4] and fusion bonding/welding [5,6,7] (in the case of thermoplastic composites). Different joining methods can also be combined in hybrid processes (using rivets, pins, or bolts) [8,9]. Mechanical joining methods are mostly known to be reliable, although they increase the weight of the structure [10]. However, these methods require holes which cut through the composite fibres and damage the composite laminate during the manufacturing process. This causes stress concentrations and local delamination, and leads to an overall degradation of the mechanical performance of the composite structure. In contrast, joining composites using adhesive bonding provides some important advantages, such as a lower process cost, high strength-to-weight ratio, low stress concentration, and a higher fatigue resistance [11,12]. Adhesive joints also distribute the load over a larger area compared to traditional joining methods [13], and therefore usually result in higher bonding strength. Moreover, adhesive bonding can be used for bonding similar and dissimilar materials and different thicknesses, which is an important advantage from an industrial standpoint, as modern structure design hinges on the combination of multiple materials with vastly different properties to optimize structure performance and cost [14].

The strength of an adhesive joint ultimately depends on the stress distribution in the bond-line and in the adherend, which is a function of different parameters such as the joint geometry and the material properties of each component (adherend or adhesive). However, it should be noted that many other parameters substantially affect the joint strength, such as the service temperature, the humidity level [15], the manufacturing process and the associated surface treatment. The mechanical properties of structural adhesives can also be effectively controlled and modified, for example, through the addition of thermoplastic compounds [16,17] or inorganic particles [18].

**Figure 1:**
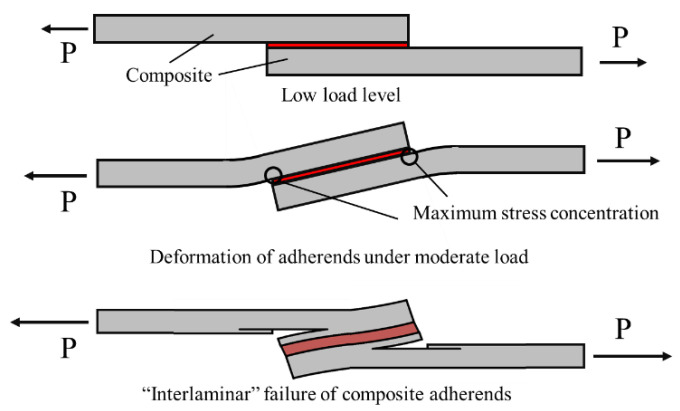
Effect of load level on the deformation and interlaminar failure of the composite adherends.

Although adhesive bonding provides an important set of advantages for bonding composites, the low interlaminar strength of composite adherends can lead to important limitations in the performance of bonded joints with composite adherends [19]. In poorly designed joints, the peel stresses generated at the bond-line can overcome the limited transverse strength of the composite and cause failure by delamination at load levels well below the strength limits of the adhesive. Figure 1 provides a schematic representation of how different load levels affect the composite adherends and eventually cause delamination.

The stiffness of the adhesive used for bonding composites is also known to be one of the key parameters which increases the likelihood of delamination failure [20,21,22]. It has been experimentally [23,24] and numerically [25,26] shown that the use of mixed adhesive joints (combining two adhesives in a single joint) reduces the local peel stresses that lead to delamination. Many other practical techniques have been proposed to prevent delamination and improve the strength of composite joints, such as the use of Z-pins [27,28], 3D weaving [29], stitching [30], braiding [31], and the use of additional thermoplastic inter-plies [32]. It should be noted that the Z-pinning method is known to be most effective, when compared to other methods, but it requires a complex manufacturing process, which increases the cost of the final product. The use of composite metal laminates [33,34] or hybrid composites [35] are other methods to improve strength of composite materials, which can increase transverse strength on the critical surface region [33] and/or result in a reduction of the shear stresses acting on the adhesive [35]. Recently, the use of hybrid bonded/riveted joints has also been found to be effective, since these joints are known to improve static strength and fatigue performance, and present higher energy absorption [9].

Multiple review papers have been published on the subject of adhesive bonding of composite substrates [8,36,37,38,39], describing a wide range of methods suitable for improving joint performance under different loading conditions. The current study further contributes to the literature by providing a detailed analysis of the recent developments on adherend modification of adhesive bonded composite joints. The effects of stacking sequence, the use of thin-plies and composite metal laminates, specific surface preparations and the use of toughened surface layers on the mechanical performance (under different loading conditions) of adhesively bonded joints have been summarized and analysed in detail.

## 2. Joint Configuration and Geometry

### 2.1. Joint Geometry

**Figure 2:**
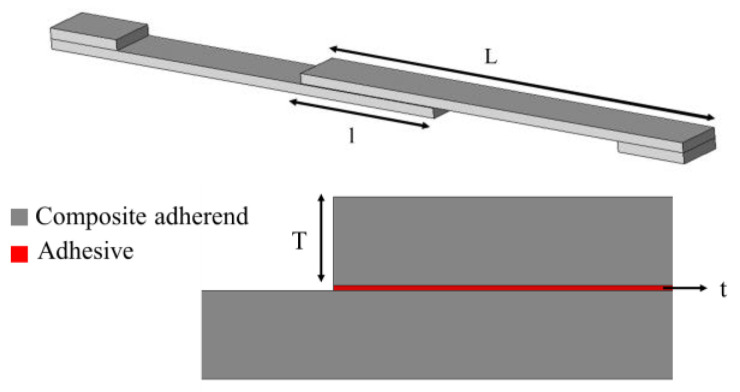
Schematic design of a single lap joint.

The mechanical performance of a bonded joint is known to be highly dependent on joint geometry, which includes factors such as overlap length, adherend and adhesive thickness, etc. Among the most significant of these parameters are the overlap length (l), adherend thickness (T), adhesive thickness (t) and the adherend and adhesive elastic modulus and shear strength [40]. Figure 2 presents a schematic design of a single lap joint, illustrating the aforementioned parameters.

**Figure 3:**
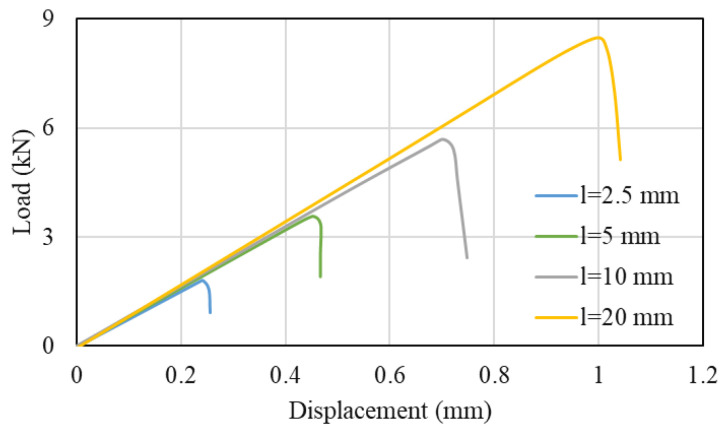
Load-displacement for different overlap length under tensile loading conditions. Adapted from [44].

Multiple research studies have been carried out to understand the effect of the overlap length in composite joints. It can be generally stated that, if the adherend does not fail or yield, an increase in the overlap length will lead to an increase in the failure load of the joint. This is known to occur even under different loading conditions e.g., under quasi-static and impact loads (see Figure 3) [41,42,43].

This increase in the failure load could be explained by the substantial effect that the overlap length has on the peel stress, a fact shown numerically by Demiral and Kadioglu [45,46]. At the same time, the failure load is also highly affected by the applied strain rate in composite joints, a result of the strain rate sensitivity exhibited by the adhesive and the polymeric resin of composites [41,44]. 

However, if stiff and brittle adhesives are used, an increase in the overlap length will result in a more modest (or even a negligible) increase of the failure load, which is in contrast to that found for joints bonded with a ductile adhesive, where the increase in failure load is found to be almost directly proportional to the increase in overlap length. This can be explained by the fact that severe stress concentrations are generated at the overlap ends by the stiff and brittle adhesive, leading to premature failure. These stress concentrations are present even for very large overlap length values, reducing the effectiveness of increasing this dimensional parameter. 

Li et al. [47] presented a thorough experimental study on the effect of adherend thickness for single lap, double lap and scarf composite joints, where a higher failure load was observed for all the mentioned configurations in larger adherend thicknesses (higher strength for single and double lap joints, but lower strength for scarf joints). In the case of scarf joints, the authors [47] showed that by increasing the scarf angle a higher lap shear strength was obtained. 

### 2.2. Effect of Surface Preparation

To ensure maximum joint strength, the bonding surfaces must be thoroughly prepared before the adhesive application, which is often a costly and time-consuming process, but essential to avoid adhesive failure. Furthermore, bond strength and durability are known to be extremely sensitive to environmental parameters such as the temperature and humidity, both of which can have a deleterious effect on the adhesive/adherend interface and degrade the level of adhesion. 

**Figure 4:**
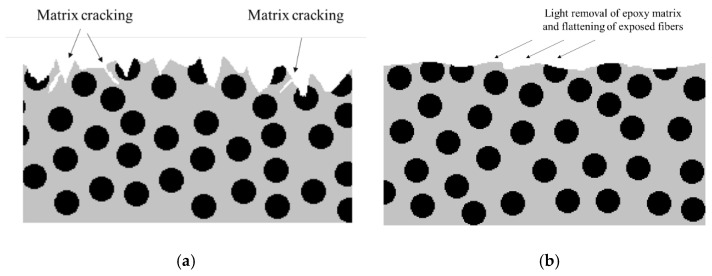
Schematic design of composite after (**a**) grit blasting and (**b**) sanding. Adapted from [72].

A proper surface treatment for composite adherends should always seek the removal of all contaminants from the surfaces and ensure a good level of adhesion, which can be achieved through an increase of wettability (increasing surface energy and the chemical activation of material surfaces being bonded) or by increasing the roughness of the surface (and increasing the level of mechanical interlocking between the adhesive and the adherend) [48,49,50]. Many different chemical and physical surface treatments are currently available for composites. These employ different methodologies, such as mechanical abrasion [51], degreasing the surface with solvent [52,53], laser ablation [54,55], plasma treatment [56,57], peel ply technique [57,58,59,60,61], irradiation [62], grit blasting [63,64] and chemical surface activation [65]. It is important to take into account the fact that the bulk mechanical properties of a composite can be strongly affected by surface treatments. For example, severe abrasive treatments can damage the composite and adversely affect joint behaviour [66,67] and bond strength [68,69]. This is because the fibres closer to the surface can be damaged by abrasion, reducing composite strength and the adhesion of the fibres to the matrix, and may even introduce contamination through loose microparticles (see Figure 4) [70]. Nonetheless, microparticles can be removed with the use of suitable high-power ultrasonic cleaning methods, and, hence, the use of primer coating together with high-power ultrasonic cleaning leads to a significant increase in strength [71].

Laser surface treatment is an eco-friendly and non-contact procedure which dispenses with harmful chemical solutions and does not introduce secondary contaminations. This method can be used to provide greater roughness and wettability, leading to higher shear strength and a cohesive failure mode when compared with conventional mechanical abrasion [51,73]. It has also been shown that aging does not significantly degrade the quality of the laser-treated interface [54]. The use of this approach is well suited for the preparation of composite adherends [74], although there is still some possibility of local fibre damage with high accumulated laser fluence [75].

The use of peel ply is a commonly used technique to protect the surface of composite laminates from contamination, while also creating and maintaining a specific surface texture [59]. A peel ply layer is used to absorb any residual resin and to create an activated surface for adhesive bonding or coating by peel ply removal. This method is also able to provide a good surface treatment for bonding purposes.

Different methods are available to assess the pre-bond quality of CFRP surfaces. One of these is the optically stimulated electron emission (OSEE) [76], able to detect weak adhesive bonds of CFRP [77], which might have been caused by contamination or poor curing of the adhesive. Other methods can be used to detect defects in the laminate itself, such as the use of electromechanical impedance [78], acoustic emission [79] or ultrasonic emission [80], or the electrical resistance method [13,81,82,83]. However, interfacial defects in the form of kissing bonds may still go undetected. Attempts using advanced ultrasonic methods such as nonlinear ultrasounds, guided waves or digital image correlation (DIC) [84] inspection to detect kissing bonds have met with limited success. However, some authors report that DIC can be effective when applied in the case of partial or localized kissing bonds [84].

### 2.3. Effect of Manufacturing Process

**Figure 5:**
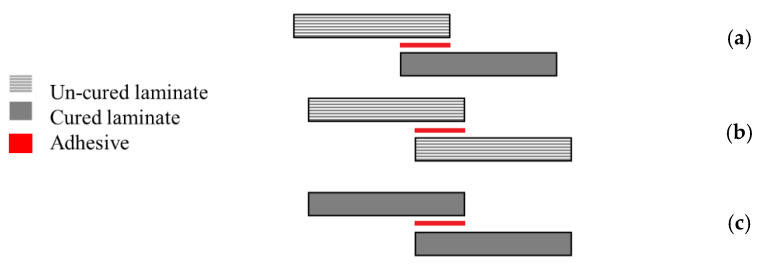
(**a**) Co-bonding, (**b**) co-curing, and (**c**) secondary bonding. Adapted from [38].

The manufacturing process of composite joints can follow three different approaches, the selection of which can be dependent on the nature of the composite and adhesive, and their curing temperatures. These processes are known as co-curing, co-bonding and the secondary bonding method [38]. A co-bonding process is performed when one adherend is cured simultaneously with the adhesive, while in the co-curing process both adherends and the adhesive are simultaneously cured. Secondary bonding is when the adhesive layer is cured between two pre-cured composite substrates [38,73,85]. Figure 5 shows a schematic design of the mentioned manufacturing methods.

Each of these manufacturing methods is known to have its own specific advantages and disadvantages [86]. For secondary bonded joints, failure is known to occur in the composite [87] and edge effects are not so predominant [22]. On the other hand, in co-bonded joints, failure typically occurs in the adhesive, since greater resistance to crack initiation and propagation has been observed [87] and significant edge effects have been observed [22]. However, the co-bonded joints have been shown to have a lower strength than the secondary bonded joints under a wide range of loading conditions [88,89,90,91]. In some cases, the moisture present in the prepreg was found to have been released during curing and had migrated to the adhesive layer, which led to a weakening of the interface and lower strength of the co-cured joints [90,92].

Notwithstanding, co-curing or co-bonding methods are usually preferred over the secondary bonding methods, because the number of parts and curing cycles needed are reduced. Hence, secondary bonding is mostly used for the repair of composite structures while for large and complex structures the secondary bonding process is more suitable [38,93]. 

### 2.4. Alternative Joint Configurations

**Figure 6:**
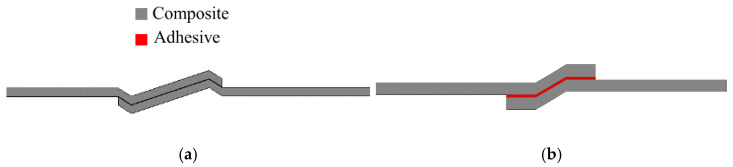
(**a**) Wavy-lap joint and (**b**) flat joggle flat joint (FJF).

As stated above, undesired peel stresses are a main cause of delamination failure in composite adherends [94]. Accordingly, a proper joint design process should seek, as an objective, to reduce the peeling stress concentration in the overlap. Many different geometrical modifications have been proposed to reduce delamination, whether through reductions of the joint overlap length [20,95], increasing the bonded area width [10] or increasing adherend thickness [2]. As an alternative, the adherend geometry can also be changed by adding tapered sections, reducing adherend thickness [23,96], using internal tapers of adhesive [97], adding a spew fillet [98,99], using adhesively bonded joint with non-flat interfaces [100] or using wavy-lap [101] (see Figure 6a). The flat joggle flat joint (FJF) [102] (seen in Figure 6b) is a relatively complex joint design which is known to reach very high failure loads due to the compressive stress field developed [101] and its ability to overcome the bending effect [102]. However, all of these modifications require cost and time-consuming machining and manufacturing steps [98]. 

**Figure 7:**
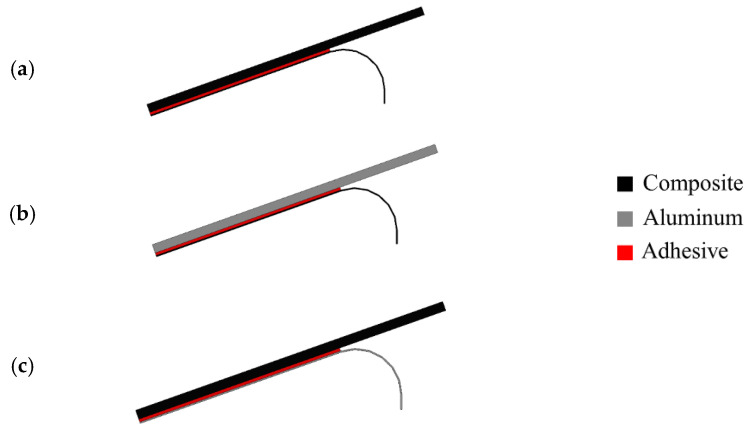
Peel tests for bonded (**a**) composite-to-composite and (**b**,**c**) composite-to-metal. Adapted from [103,104].

A study from Teixeira and Sinke [103] showed that peel tests could be performed in bonded composite-to-metal and composite-to-composite joints using a floated rolling peel test (see Figure 7) [103,104], and concluded that using CFRP as the flexible adherend has a considerable effect on the peel load since the peel load gives a direct indication of the failure mode [103]. 

In conclusion, the use of modifications in joint configuration has been shown to be a powerful method for overcoming the bending effect typical of unbalanced joints, and it can also be used to develop a compressing stress field in the overlap length. Moreover, the literature demonstrates that delamination can be eliminated or delayed, and joint strength can be improved in a composite joint, by modifying the shape and configuration of the adherend or the adhesive layer. This issue is discussed in further detail in Section 3 and Section 4.

## 3. Adhesive Layer Modifications

### 3.1. Mixed and Functionally Graded Adhesive Layer

In adhesive joints, failure is usually the result of a non-uniform distribution of stresses in the bond-line [105]. A non-uniform stress distribution is even more obvious in joints with dissimilar adherends and those operating in an extreme temperature range [106]. In composite joints, whether with similar or dissimilar adherends, the stiffness of the adhesive used for bonding composites is known to be one of the key parameters controlling the onset of delamination, with several authors demonstrating that low strength yet flexible adhesives are able to outperform stronger but stiffer adhesives [20]. 

**Figure 8:**
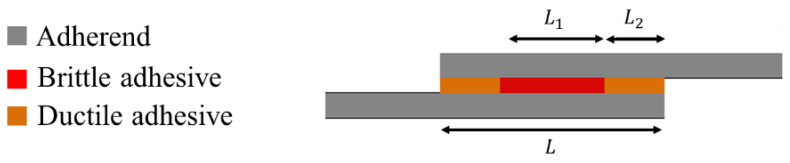
Mixed adhesive joint.

Mixed adhesive joints, in which two different adhesives (one ductile and one brittle adhesive) are used along the bond-line, are known to be a valid method for avoiding the formation of non-uniform stress distribution in a bond-line. In this approach, the brittle adhesive is utilized in the middle part of the bond-line and the ductile adhesive is used at overlap ends, where higher stress concentrations occur (see Figure 8) [107,108]. The dual adhesive concept is also a viable alternative for cases where there is a large difference in the properties of two dissimilar adherends and where the joint must operate under demanding environmental conditions [109].

**Figure 9:**
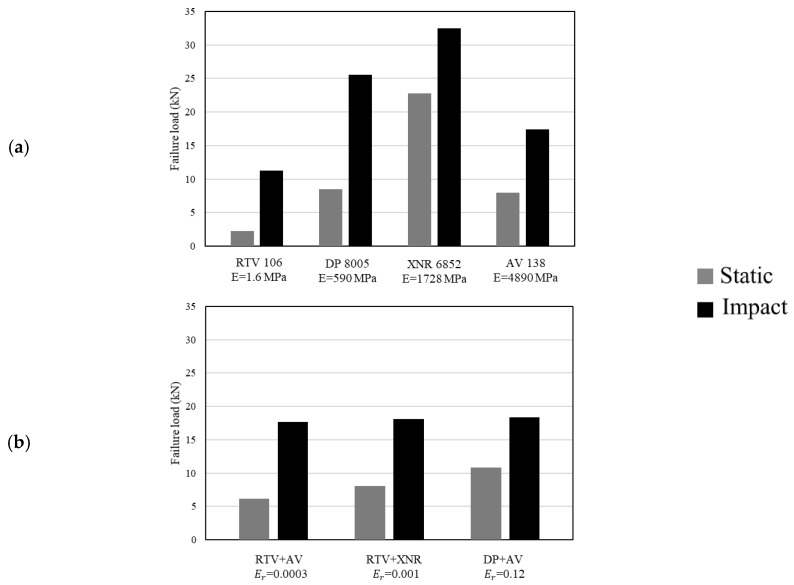
Failure load for single and mixed adhesive joint for (**a**) single adhesive (E represents the adhesive’s Young’s modulus) and (**b**) mixed adhesive joint (E_r_ represents the ratio of flexible adhesive’s Young’s modulus to stiff adhesive’s Young’s modulus). Adapted from [111].

Generally speaking, studies on structural adhesive joints for bonding aluminium with composite materials have led to the conclusion that the most suitable adhesives for use in these configurations are toughened epoxies (where stiffness is indispensable) and polyurethanes (for dynamic mechanical requirements that call for flexibility) [110]. Logically, these materials are also suitable for combination in a mixed adhesive joint with composite substrates. Nonetheless, it is worth mentioning that the performance of a mixed adhesive joint under different loading and testing conditions (quasi-static and impact) is highly dependent on the material properties of the adhesives used [24]. Adhesive selection is crucial for maximizing not only the performance of the bond-line but also that of the adherents. The work of Machado et al. [111] demonstrated this by studying the use of four different adhesives in a mixed-adhesive single lap joint configuration (AV 138 and XNR 6852 were used as stiff adhesives and DP 8005 and RTV 106 as flexible adhesives). According to the results, presented in Figure 9, for both quasi-static and impact loading conditions, the use of a mixed adhesive joint instead of single adhesive layers does not always guarantee an improvement of the shear strength, and this is especially evident when the performance (under different loading conditions) of mixed joints is compared with the use of a brittle and ductile single adhesive joint. 

**Figure 10:**
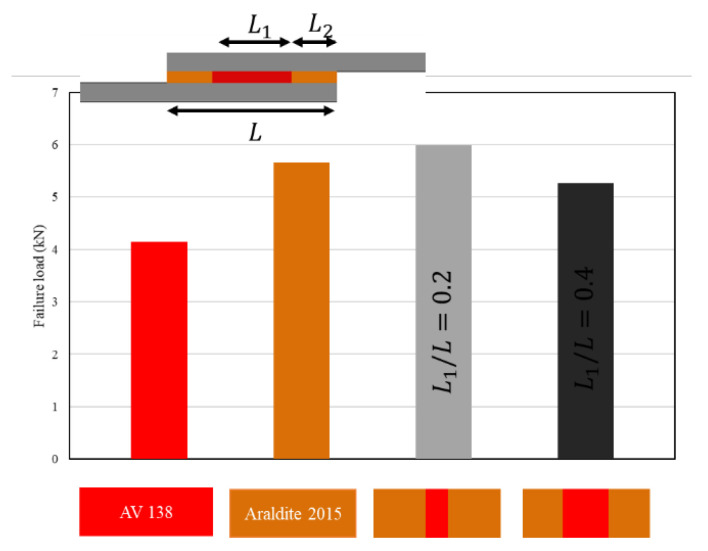
Effect of length ratio on failure load in mixed adhesive joint. Adapted from [109].

**Figure 11:**
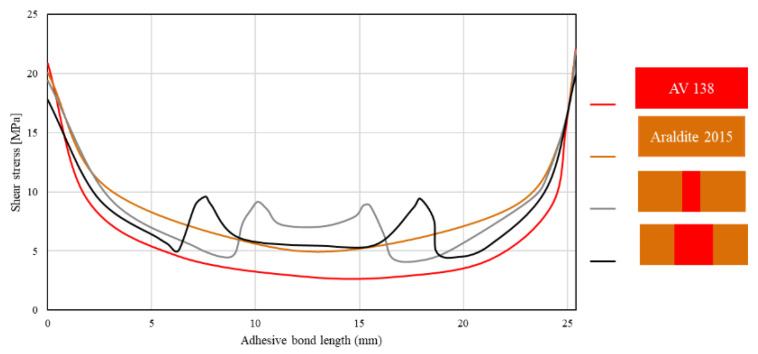
Effect of length ratio on shear stress distribution in mixed adhesive joint. Adapted from [109].

Jairaja and Naik [109] studied two configurations for dual adhesive joints, including 20% and 40% overlap length for the brittle adhesive (L_1_/L). They used two different adhesives, AV 138 (as the brittle adhesive) and Araldite 2015 (as the ductile adhesive), in a single lap joint with dissimilar adherends. As shown in Figure 10, experimental results have demonstrated that a length ratio of 0.2 presents the highest failure load over both brittle and ductile reference single lap joints. Furthermore, the configuration with the length ratio of 0.4 presents a lower failure load as compared to the single lap joint with ductile adhesive. A parallel numerical analysis was performed, in which it was observed that the use of dual adhesive layers presented lower stress concentration at the overlap edges (see Figure 11). The mixed adhesive bond-line with 20% of brittle adhesive presented a higher bond strength than single adhesive joints using brittle and ductile adhesives. The mixed adhesive bond-line with 40% of brittle adhesive presented lower strength when compared to the ductile adhesive. 

**Figure 12:**
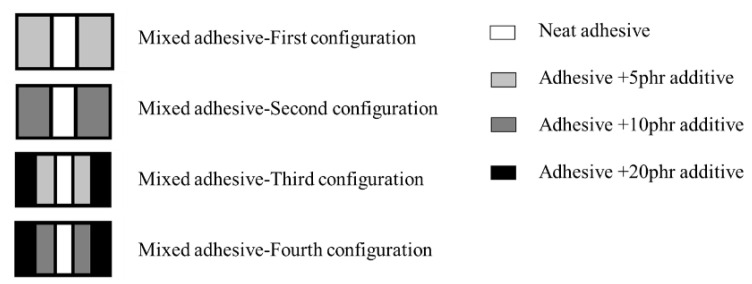
Schematic design of mixed adhesive joint studied by Dadian and Rahnama [105].

Functionally graded adhesive layers can be seen as a natural evolution of mixed adhesive layers in which the material property of the adhesive changes gradually rather than discretely (see Figure 12). These are considered as highly effective, but hard to implement alternatives to reduce the peel stress concentrations located at bond-line ends [112,113]. Dadian and Rahnama [105] performed an experimental and numerical study on functionally graded joints with dissimilar adherends, where 7075-T6 aluminium and a CFRP adherends were used. This research used a neat adhesive and four other epoxies containing different amounts of additives, namely 5,10, 15 and 20 phr (parts per hundred rubber), which led to adhesive formulations with increasingly higher ductility. 

**Figure 13:**
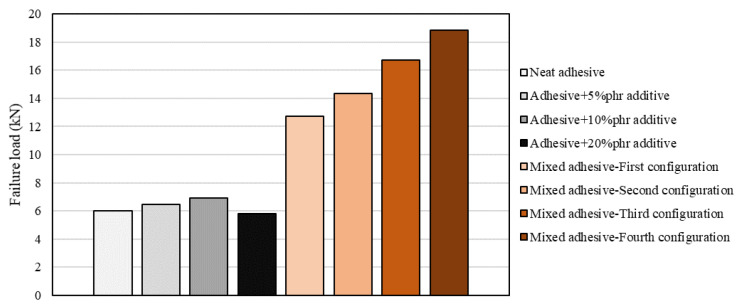
Effect of mixed and functionally graded adhesive layer. Adapted from [105].

Ductile adhesives were used at the edges of the overlap and the number of bands in the overlap was increased by adding adhesives with intermediate properties between two adjacent adhesives. As a result, the stress concentrations at both edges decreased and the inner zones of the adhesive layer were now able to provide a larger contribution to the overall load-bearing capacity of the joint. As seen in Figure 13, shear strength was increased significantly in a mixed adhesive configuration, especially when compared to the related brittle and ductile adhesive [105]. It should be mentioned the failure load increases as the number of bands in the overlap increases.

Considering the works analysed in this section, one can state that the use of mixed adhesive bonded joints has already a good track record in achieving strength improvement of composite joints. However, this technique hinges on fine balances, as the material properties of the adhesives (brittle and ductile) and their relative dimensions require careful optimization in order to achieve the best results. Moreover, the use of functionally graded adhesive joints is even more advantageous when compared to a mixed adhesive bonded joint, but it is beset with significant manufacturability issues.

### 3.2. Nano-Reinforced Adhesive Layers 

**Figure 14:**
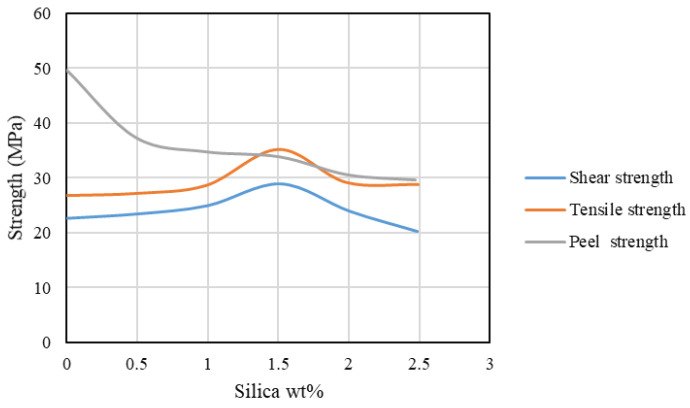
Effect of filler content on joint strength. Adapted from [117].

Adhesive layers modified with the use of nanoparticles have been a recent topic of interest [114], being based on research that has shown nanocomposites to exhibit much better mechanical, thermal, and barrier properties than do polymer-based composites [115]. The failure load of a nano-reinforced joint is significantly affected by multiple parameters, such as whether the adhesive is rigid, flexible, or toughened and the ratio and type of the added nanostructure [116]. Further studies have shown that the addition of a small amount of nanoparticles to the adhesive, at as low a ratio as 1–1.5%, often results in a drastic improvement in its properties [12,116,117,118], with direct effects on the mechanical strength of structural joints [119] both in the shear and tensile loading modes [117,120,121,122,123]. This behaviour is the result of a more efficient stress transfer between nanoparticles and polymer matrix, which improves the cohesive properties of the bond. However, some works report a decrease in the peel strength of the bonded joints [117], attributed to an increase in the glass transition temperature (T_g_) and the increased brittleness of the adhesives with higher nanofiller content. The addition of nanoparticles improves the interfacial wettability of the substrates (composite or metal) [117,124], and it is known to be the reason behind drastic shifts in the failure mode [125], which changes from interfacial failure, with no significant damage on the composite adherends, to cohesive failure in the adhesives, where the load is more effectively transferred to the adherends [12]. It has also been reported that, due to their small dimensions, nanofillers can penetrate into small voids on the adherend’s surface, allowing for the joint strength to be enhanced via improved mechanical interlocking [126]. Furthermore, fracture surfaces also seem to be strongly affected by the addition of nanoparticles, often transitioning from a relatively smooth surface to a rougher and grooved morphology [117,124]. In practice, this suggests that more energy is needed to break the material if an optimal amount of nanoparticles is used [124]. The definition of this optimal value is where the main challenge of using these reinforcements lies, since the addition of particles above a given value will eventually result in a composite joint with reduced static performance (see Figure 14) [12,116,117]. This limit can be attributed to the eventual formation of poorly connected material agglomerations in specimens with higher amount of the filler content [12,117] or incompatibilities between the particles and the adherend surfaces and adhesives [12]. 

Carbon nanotubes (CNTs) are being widely used as reinforcement nanofillers in polymer nanocomposites and are categorized as single-, double-, or multi-walled, based on the number of concentric graphene sheets rolled together to make up the nanotube. Multi-wall carbon nanotubes (MWCNTs) are often employed as reinforcing nanofillers in composite materials, which results in materials with high strength and stiffness [11,127]. MWCNTs can also be used to reinforce adhesives [18,116] and have been shown to be an effective alternative for improving mechanical (e.g., toughness, strength stiffness, and fracture energy), electrical, and thermal properties for multiple applications [127,128,129,130,131]. Improvements in adhesion can also be expected, as reductions in the contact angle have been reported as a result of the inclusion of a low content of MWCNTs in epoxy [129]. Additionally, the presence of MWCNTs in adhesives results in the enhancement of the resistance to crack formation and propagation [11]. These materials can also potentiate crack bridging, and act as a barrier in the crack propagation path [127]. This happens because crack initiation and propagation times are generally larger when carbon nanotubes are dispersed in adhesives [132]. In a similar manner, the use of these materials has also been shown to result in increased fatigue performance [132,133]. 

A nanotube reinforcement process can have many different characteristics, such as dispersion, structure, and nanotube length and diameter. Research has found that all these characteristics play an important role in the static performance of the reinforced joint [120]. Although this is still an incipient field of research, experimental results obtained so far show that the addition of shorter and thin nanotubes generally results in weaker bonding in the composite–composite joint tests [3]. Some authors further postulate that the optimum nanotube reinforcement should be around 1% [3,120], and that an extra addition of nanotubes leads to a general decrease in the lap shear strength [81]. It has also been shown that the use of CNT opens the door for advanced interfacial damage detection. A highly conductive CNT network will show noticeable changes in electric resistance as the crack grows [133,134].

## 4. Substrate Modifications

**Figure 15:**
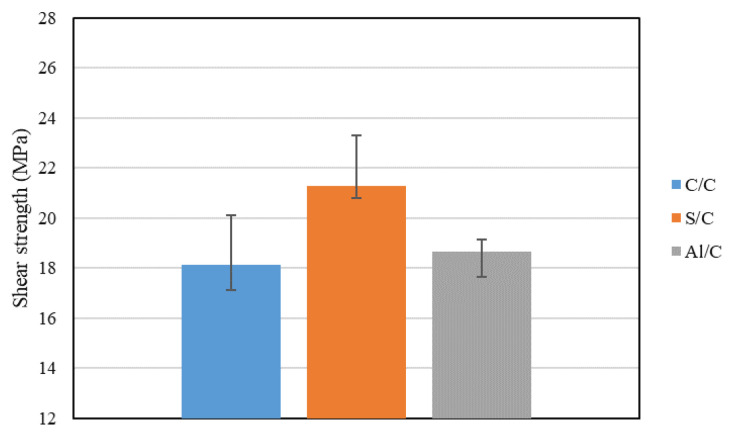
Effect of dissimilar adherend on single lap joint shear strength. Adapted from [42].

Due to the significant growth in the use of composites, such as CFRP, and the limited number of processes available for joining these materials, adhesive bonding is an integral part of composite design. In light of this, research has been carried out on the modification and adjustment of composite properties, seeking to optimize the joint performance in a holistic manner. Different methods have been found to have positive influence on strength, e.g., by increasing the rigidity of the adherends [42]. This could be achieved by modifying both of the adherends, or at least one, to minimize the rotation of the joint and promote a more uniform distribution of stresses in the adhesive. As shown in Figure 15, by replacing one of the composite adherends with a metallic material (in this case the joints under analysis combined steel/composite (S/C) and aluminium/composite (Al/C), higher strength could be attained than joints with symmetrical composite/composite (C/C) adherends. 

**Figure 16:**
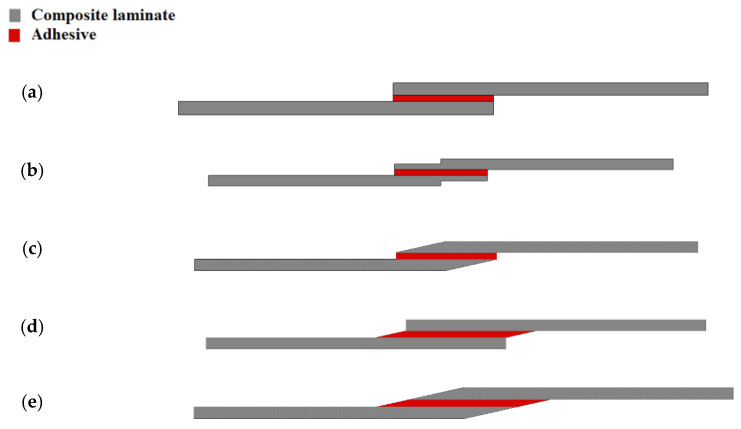
Schematic design for (**a**) single lap joint, (**b**) adherend recessing, (**c**) adherend chamfering, (**d**) adhesive chamfering and (**e**) adherend and adhesive chamfering. Adapted from [136].

Another approach consists of locally recessing the adherends [135] (see Figure 16b), which creates a peak of the peel stress in the adhesive at the point where the recess was made, but one that is lower than the peak peel stress at the edge of the overlap, and, thus allows for a more uniform use of the available overlap length [136]. In this case, the depth of the recess seems to be the most effective parameter and not the length [136]. The effect of chamfering was numerically studied by Moya Sanz [136], including the effect of the chamfer angle upon the adherend, the adhesive or both. Schematic designs of this configuration are shown in Figure 16c–e), respectively. The best-performing configuration had both adherends and the adhesive chamfered at an angle of 15%.

### 4.1. Effect of Stacking Sequence

**Figure 17:**
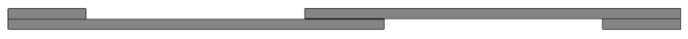
Single lap joint studied by Ozel et al. [43].

**Figure 18:**
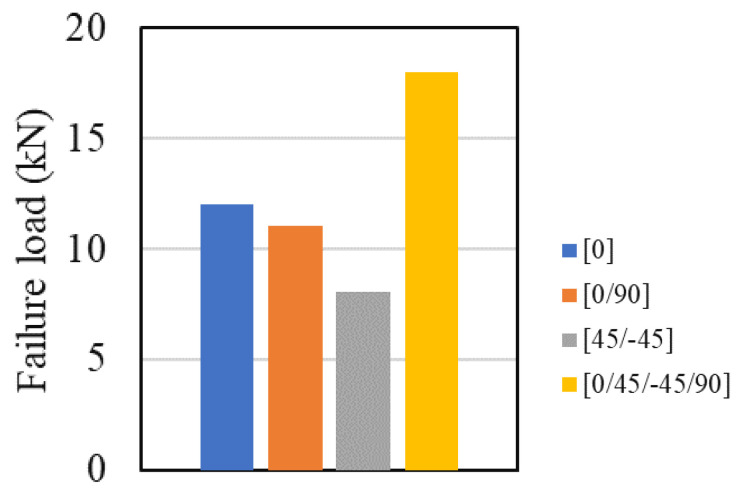
Effect of stacking sequence on failure load composite single lap joint. Adapted from [43].

Although there are many different techniques that allow for increased performance (under different loading conditions) of bonded joints, in composite joints, stacking sequence of the adherends is a uniquely effective parameter and, thus, has been the target of extensive study. It is worth noting that the effect of the stacking sequence is highly dependent on the joint configuration and on the material properties and that the optimized stacking sequence can be varied by changing these parameters. Ostapiuk and Bienia [137] used composite material, particularly CFRP or GFRP (glass fibre reinforced plastic), to connect aluminium adherends and consequently studied two different stacking sequences, [0] and [±45] for the composite part of a composite metal laminate single lap joint under quasi-static loading. Their results showed that regardless of the composite type and the surface preparation, the [±45] presented the highest failure load. This may be due to the more complex crack path expected for an initiated crack in the angle-plied composite. Ozel et al. [43] studied four different stacking sequences in composite single lap joints ([0],[0/90], [45/−45] and [0/45/−45/90]) in which both adherends (see Figure 17) were composites. As shown in Figure 18, the [0]_16_ stacking sequence was found to present a higher failure load than the angle-plied configurations, except for the [0/45/−45/90] layup. A related numerical study determined this to be due to the low peel stresses acting on the overlap edges in the adherends with quasi-isotropic stacking sequence. The same consideration is also applicable here, explaining why, in a quasi-isotropic stacking sequence, the expected crack path for an initiated crack in the addends is more complex. Demiral and Kadioglu [45] have shown that, with the increase of fibre orientation angle, the failure mode changes from interfacial failure towards delamination in the composite adherend.

**Figure 19:**
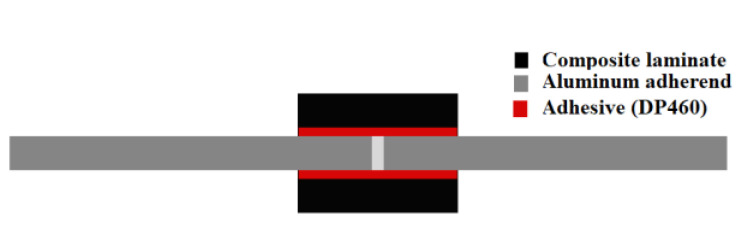
Double strapped composite joint as studied by Akpinar [138].

Akpinar [138] studied the effect of five different orientations ([0]_16_, [88]_16_, [0/90]_8_, [45/−45]_8_, [0/45/−45/90]_4_) in the static performance of composite double strapped joints under tensile loading (see Figure 19), and concluded that joints with composite patches of [0/45/−45/90]_4_ presented the highest failure load of all configurations under study. Furthermore, angle ply laminates generally presented higher failure load than all unidirectional layups, except for [88]_16_. The same was observed for double lap joints tested under impact loads [139]. The lowest shear strength was recorded for the joints having substrates with fibres oriented perpendicularly to the impact loading direction. 

**Figure 20:**
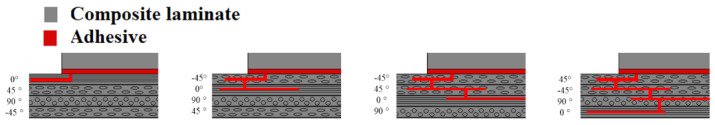
Effect of 0° layer distance from surface in quasi isotropic laminate. Adapted from [140].

The maximum shear stress was higher when dissimilar adherends were used (with different stacking sequences), which is due to the loss of homogeneity caused by the imbalance between the two adherends, combined with the edge effects of their discontinuities [139]. Purimpat et al. [140] showed that the strength of the specimens is dependent on both the local orientations and the global properties of the laminates. A vast study was performed on quasi-isotropic quasi-homogeneous (QIQH) sequences and it was concluded that, since it is more probable for the final failure to occur in the 0° layer (seat of the final break), it can be assumed that its distance from the adhesive layer increases the complexity of the crack path and thus raises the joint strength [140]. This can be seen schematically in Figure 20.

In general, the optimum stacking sequence is highly dependent on the joint configuration. However, stacking sequences with unidirectional fibres perpendicular to the loading direction and quasi-isotropic stacking sequence tend to perform better for many common joint configurations, such as the single lap joint.

### 4.2. Thin-Ply Laminates

The use of thin-ply laminates is a relatively recent approach for substrate modification. Thin-ply laminates are defined as those composed by plies with a thickness of less than 100 μm [141,142]. These layer thicknesses became available through recent developments of the spread-tow process [143], one which produces flat, straight plies until a dry ply thickness as low as 20 μm is reached [144]. Failure strength and ultimate strength of laminates can be greatly improved with a significant decrease of the fibre areal weight [145], The non-monotonic strength change of thin-ply laminates is ultimately brought on by two competing mechanisms. The decrease of ply thickness increases the specific strength while the decrease of fibre volume fraction leads to some reduction in strength [20].

The use of thin-ply laminates brings a higher degree of freedom to layup design (both in orientation and the quantity of the individual layers) [146]. Furthermore, due to the reduced layer thicknesses and the improved resin spreading process, more homogeneous fibre distribution and smaller resin-rich regions can be achieved [145]. The higher number of layers and the associated higher number of interfaces also causes the shear stresses to be lower [146], and thinner plies are acknowledged to have higher resistance against matrix cracking [147,148]. Currently, the use of thin-ply laminates is mainly driven by the search for enhanced static mechanical performance [141] as well as the ability to suppress transverse microcracking [146] and free edge delamination [147,148,149,150]. This last failure mode is a function of ply thickness, as well as the stacking sequence [151]) for quasi-static, fatigue and impact loadings [143] and is typically observed in conventional composite materials [146]. The crack suppression effect may be caused by a decrease in the energy release rate at the crack tip in the thin layer [152]. Additionally, thin-plies have other unique advantages, such as higher in-situ transverse strength. The theory of in-situ strength was proposed by Camanho et al. [153], to demonstrate that a decrease in ply thickness can be correlated to an in-situ effect, characterized by a reduction in the applied stress needed to extend a transverse crack, along the thickness of the ply, when the ply thickness increases. Based on Camanho’s ply failure criteria, damage onset in the composite is likely to occur at the same load level as in the adhesive in the case of the thick configuration and at 50% higher load level in the case of the thin configuration [154].

Kupski et.al [154] studied three different single lap joint (SLJ) configurations using thin-plies, namely: [45_4_/0_4_/−45_4_/90_4_]_S_, [45_2_/0_2_/−45_2_/90_2_]_2S_ and [45/0/−45/90]_4S_ by resorting to NTPT-HTS(12K)-5–35% prepreg, which is a thermoplastic-toughened epoxy resin with an unidirectional prepreg system. The thickness of a single ply was 50 μm, and all adherend laminates were manufactured with 32 layers of a single ply adding up to 1.6 mm of total adherend thickness. Experimental results showed that, with decreasing ply thickness, damage initiation was postponed to higher load levels, although it resulted in a more sudden damage progression until the final failure [154]. In the end, an increase in the SLJ shear strength and in the strain energy was obtained [154], and the numerical study indicated that with decreasing ply thickness, the damage onset moves away from the adhesive interface towards the mid-thickness of the adherend [154].

### 4.3. Composite Metal Laminates

**Figure 21:**
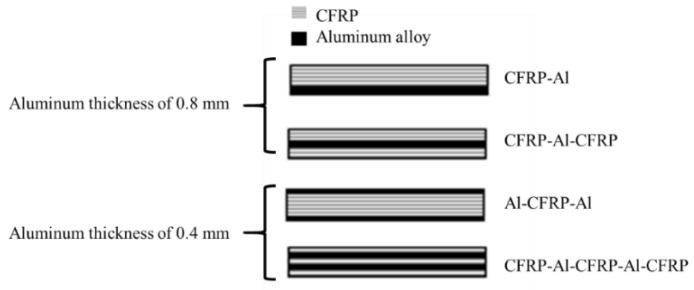
CML configurations studied by Carbas et al. [155].

Composites are structurally more efficient than metals, although the latter have better damage tolerance and fail in a more predictable manner. Metals are also generally unaffected by the solvents and temperature levels which readily degrade polymers [38]. Therefore, in order to optimize the benefits provided by both types of materials (in what regards to the strength, weight and durability of structures), a combination of traditional metals with composite materials has been pursued in recent years [38]. These materials are typically known as composite metal laminates (CMLs) and were initially developed for applications in the aerospace industry. These materials consist of metal and composite layers, and different configuration of composite metal laminates can be used as adherend in a single lap joint, as it can be observed in Figure 21. Carbas et al. [155] showed that the strength of hybrid joints can be increased when thin aluminium sheets are placed in the outer layers of the lay-up. The aluminium sheets serve as a local reinforcement, being able to prevent delamination and increased strength over the CFRP-only joints. For overlap lengths of 50 mm, hybrid joints that had aluminium in the middle of the layup exhibited just slightly higher strength values than the reference joints.

**Figure 22:**
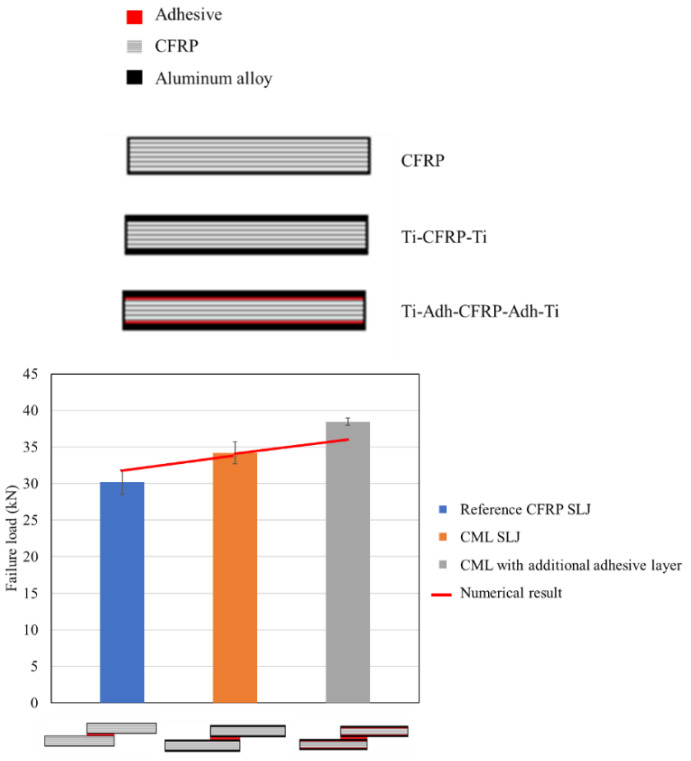
Failure load obtained by Santos et al. [158] for CML and CMLs with additional adhesive layers.

In a subsequent work, Ramezani et al. [156] replaced layers of prepreg with aluminium plates on the outer surfaces of each adherend (CML) and subsequently studied the effect of the aluminium plate thickness, while maintaining the overall thickness of the adherend constant. The experimental results show that replacing layers of CFRP with metal laminates increases the failure load under quasi-static [156], intermediate [156] and impact testing rates [157]. This can be explained by the minimization of the stress concentrations at the edges of the overlap, as the compliant and tough metal plate is able to redistribute stresses over a much larger area without any failure. In a related work, Santos et al. [158] studied novel single lap joints of CFRP joints with composite metal laminates and additional adhesive layers. As shown in Figure 22, experimental results showed an increase in the novel single lap joints compared to the ones without an additional adhesive layer.

**Figure 23:**
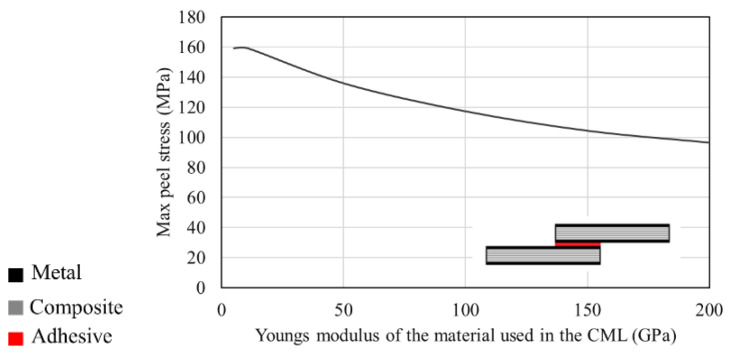
Effect of the Young’s modulus of the metal laminate used in CML on maximum peel stress. Adapted from [158].

Considering that these materials were initially designed for aerospace applications, one important component of the material selection process was to balance the stresses present on the structures. As seen in Figure 23, for a CML single lap joint, the maximum peel stress induced in the joint is reversely affected by the Young’s modulus of the metal laminate. [158]. 

**Figure 24:**
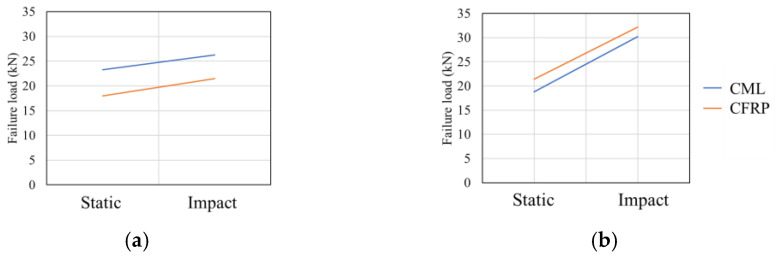
Failure load under quasi-static and impact loading for (**a**) AF 163-2K and (**b**) XNR 6852 E-3. Adapted from [157].

Morgado et al. [157] showed that the static and impact performance of composite metal laminates is highly dependent on the material property of the adhesive under both quasi-static and impact loads. As seen in Figure 24, the hybrid joint presents higher shear strength while using AF 163-2K adhesive (epoxy-based structural adhesives in film form) in both quasi-static and impact loading, which contrasts with what was observed while using XNR 6852 E-3 adhesive (epoxy-based paste adhesive). The same trend was observed by Carbas et al. [159], where the effect of reinforced hybrid composite single lap joint was also determined to be highly dependent on the adhesive. The authors found that the adhesive with lower stiffness behaved better than stronger and stiffer adhesives in regard to delamination prevention.

In the case of the adhesive with lower stiffness, higher joint strength is found for both quasi-static and fatigue loadings [103,157]. Thus, while adhesives with high tensile strength might appear more attractive for use in the assembly of high performance (static and fatigue) composite structures, this does not always translate into high joint strength [20,21,22]. In fact, the inherent stiffness of these adhesives often results in higher peel stress at the interface, leading to premature failure by delamination [157].

Overall, CMLs have been experimentally shown to be a good alternative for use with conventional composite laminates, leading to bonded joints which perform well under diverse loading conditions. The material properties of the metal laminate being used significantly impact the stress distribution in the joint and thus directly affect the joint strength. However, selection of the adhesive is ultimately the most critical factor since excessive stiffness might generate high peel stresses which wholly suppress the effect of adding the metal layer.

### 4.4. Toughened Surface Layers

**Figure 25:**
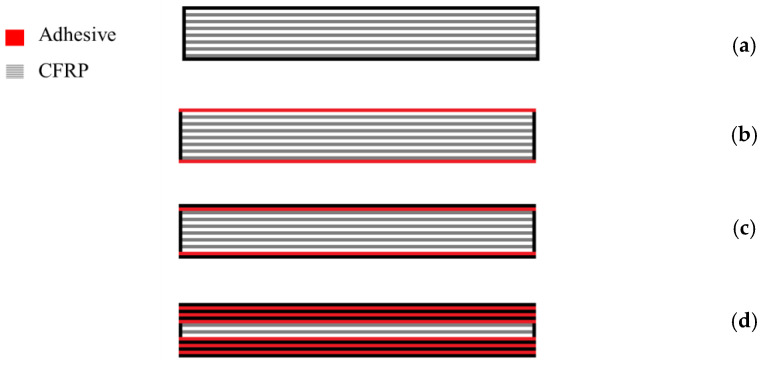
Schematic design of (**a**) CFRP, (**b**) a single external adhesive layer, (**c**) a single interlaminar adhesive layer, and (**d**) three interlaminar adhesive layers. Adapted from [156,160].

The use of toughened, non-metallic, surface layers in composite layups represents another alternative for improving the performance of bonded composite joints under different loading conditions. In this method, a high toughness and compliant layer is applied to both outer surfaces of the composite material, one which will serve as an adherend in a bonded joint. The toughened layer could take the form of a non-reinforced resin or a less stiff fibre reinforced composite material. The use of toughened adherends is known to delay [156] or even completely eliminate [125] delamination failure in joints with composite substrates. Different possible configurations of composites toughened with polymers layers are schematically presented in Figure 25.

Morgado et al. [160] evaluated the use of a single interlaminar adhesive layer, three interlaminar adhesive layers and a single external adhesive layer, which together is known as adhesive layer reinforcement (ALR). Under quasi-static loads, all the optimized designs under evaluation performed better than the reference CFRP-only joint. The configuration with external adhesive layers exhibited the highest strength increase, raising 23% above the failure load of the reference CFRP-only joint. Most importantly, the failure mode changed from delamination to cohesive failure of the adhesive itself, while the reinforcement layer remained fully intact [156,160].

**Figure 26:**
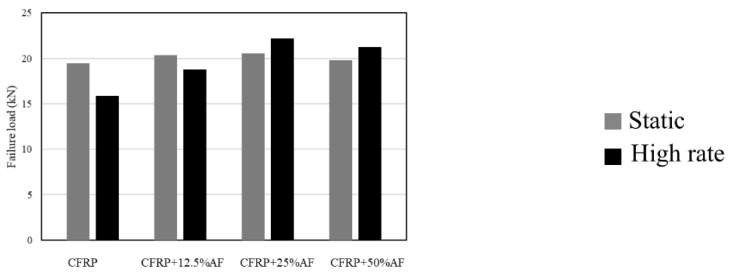
Failure load obtained for composite single lap joints with toughened adherends. Adapted from [156].

Experimental studies have demonstrated that the use of toughened adherends results in an enhancement of the shear strength of bonded joints [125] loaded under different testing rates (quasi-static, intermediate rate and impact loading) [156,160]. However, the authors note that an optimum thickness of toughened material is dependent on the specific characteristics of the adherend to avoid drastic decreases of the joint strength [156]. Figure 26 presents the failure load of bonded joints created using a reference CFRP adherend and the toughened hybrid single lap joints obtained by Ramezani et al. [156]. The results clearly show that the use of toughened layers in composite joints leads to an effective increase in joint strength. This increase is the result of two different factors. The first is the increased loadbearing capability provided by yielding of the toughened surface layer before failure occurs [125]. The second factor is associated to the lowered stiffness of the surface toughening material, which is usually much lower than that of the base composite material and thus reduces the presence of stress concentrations at the edges of the overlap length of the bonded joint [156].

Shang et al. [125] studied the use of novel composite material as an adherend, one composed of 0.5 mm thick layers of glass fabric reinforced on both surfaces and a 1 mm thick CFRP core. The novel composite adherend was found out to have 22% higher failure load than that of specimens using CFRP-only adherends. Again, the failure mode was found to change from delamination of the adherends to cohesive failure in the adhesive, which was ultimately the main target of this work.

**Figure 27:**
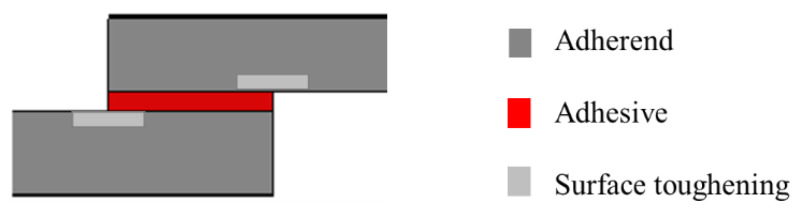
Localised surface toughening concept. Adapted from [161].

Schollerer et al. [161] studied a novel local adherend surface toughening concept by using a localized thermoplastic layer, as shown in Figure 27. An increase of up to 84% in the failure was observed to be the result of localized surface toughening in joints. The toughened surfaces create less peel and shear stress concentration in the bond-line ends. It should be noted that an optimum length of surface toughening was found to exist, whereupon the use of a reinforcement larger than the optimum surface toughening length results in adherend delamination.

In light of these results, while a toughened composite laminate with external adhesive layer (Figure 25b) presents the highest strength among all other comparable configurations, the use of composite adherends with toughened surfaces is now seen as a very promising method to increase joint strength and to delay or eliminate delamination in adhesive joints intended for service under for different loading conditions.

## 5. Discussion

**Figure 28:**
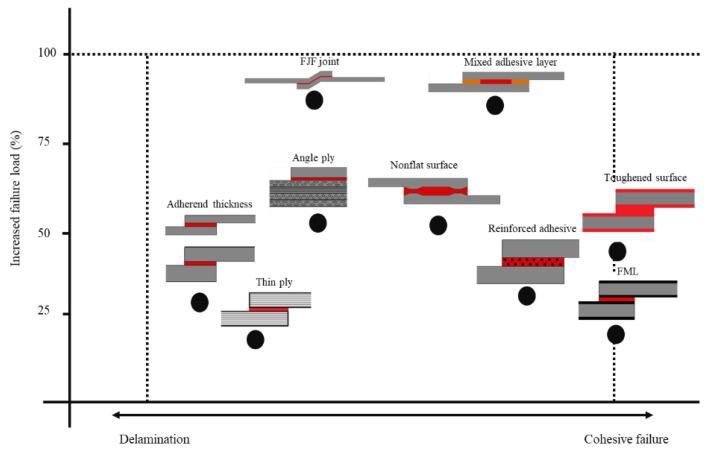
A comparison of different techniques in terms of strength improvement and failure mechanism.

An analysis and discussion of the results described in the literature allows for a relative comparison of the different revised techniques both in terms of strength improvement and in their failure mechanisms. To this aim, a scheme showing a comparison between the basic characteristics of the different processes is presented in Figure 28. Considering non-modified joints with CFRP adherends as a reference, an ideal reinforcement technique would allow for a substantial increase in failure load, while avoiding delamination (by providing fully cohesive failure in the adhesive layer).

It is known that changes in the joint geometry, specifically the substrate thickness, allow for increases in the failure load above 25%. However, higher improvements are limited by the onset of delamination. A similar phenomenon is also observed when thin-plies are introduced in composite laminates. Even though this technique allows the delay of crack initiation and propagation, delamination failure will almost always eventually occur, precluding the adhesive layer from attaining its maximum potential. However, the use of different fibre orientations-angle plies, does allow the joint to achieve more significant improvements to its performance, both in terms of strength and failure mode. Further improvements can be attained only with more complex joint configurations, such as those using highly shaped adherends and reinforced adhesives. The former possesses geometrical features which are specially designed to overcome the bending effect and to create compression states, all of which serve to reduce the peel stresses that limit the joint’s performance. On the other hand, modifications of the adhesive are also possible, such as those which rely on the use of nanoparticles, which improve stress transfer in the adhesive layer. Full cohesive failure has only been demonstrated for the case of two types of adherend modifications: CMLs and the use of toughened surface layers. The metal-composite hybrid approach creates an improved load transfer platform from the adhesive to the metal that although it has to withstand considerable stresses, makes use of the high toughness and compliance of metals to avoid damage. Adherends reinforced with toughened layers, exhibiting a failure mechanism similar to that observed in CMLs, take advantage of the properties of the resin used, creating highly ductile and compliant surface layers that improve stress distributions and avoid delamination in the substrate. From the strength improvement perspective, the two geometries that have been shown to have superior static performance are the FJF joints and joints using mixed adhesive layers. All of the configurations described above, regardless of their failure mode, lead to an increase in strength that varies approximately between 20–60%, but FJF joints can show an increase of almost 100%. FJF joints take advantage of their geometric configuration to guarantee that compression stresses delay joint failure. However, the presence of notches in the composites can also induce localized delamination. In mixed adhesive layers, performance is maximised through a precise control of adhesive stiffness and thus of the peel stress distribution. While these solutions are admittedly technically complex, they have been shown to be able to overcome non-uniform distribution of the adhesive layer, increasing its strength.

Although multiple solutions have been proposed presented and demonstrated to maximize the performance of composite bonded joints, determining an optimal solution for practical applications is still a significant challenge. Nonetheless, a simple conclusion can be extracted from this analysis. Irrespective of the solution chosen, the ideal joint should always be designed to minimise peel stresses at the edge of the overlap, avoiding delamination, but without damaging the substrates through time-consuming and complex geometric alterations, while meeting the practicality demands associated with cost and manufacturability concerns. An efficient load transfer, with a customised distribution of adhesive stiffness, may be the basis of a solution closer to the ideal.

## 6. Conclusions

The present work reviews recent developments in adhesively bonded composite joints, with particular interest in adherend modification techniques. The effects of stacking sequence, the use of thin-ply, composite metal laminates and its specific surface preparations, and the use of toughened surface layers in the composite adherends were thoroughly analysed in the context of adhesively bonded CFRP joints and structures.

- It has been shown that, optimising the joint geometry, e.g., the substrate thickness, can cause an increase in the failure load, although substrate delamination will still be a concern. Advanced joint configurations were proposed for composite joints, being shown that, among all configurations under analysis, the FJF joints exhibit the highest static performance, but are still susceptible to delamination due to the modifications that must be made to the composite.
- The adhesive layer can be modified by using a functionally graded or a nano-reinforced adhesive layer. The former overcomes non-uniform distribution of the adhesive layer and allows for the delay of delamination failure. The latter creates efficient stress transfer between nanoparticles and the polymer matrix, which improves the strength of the bond.
- The use of different fibre orientations does allow the joint to improve its performance and strength, forcing the crack to grow through more complex crack paths.
- The use of thin-plies in laminates can cause a delay in crack initiation and propagation due to lowered ply thickness and increased fibre ratios. In addition, the higher degree of design freedom they provide allows for the use of a wider range different fibre orientations and leads to an increase in the static performance of the joint. However, there are very few papers studying the effect of using of thin-plies in composite joints and this technique has now been shown as a viable concept for future research and developments.
- Fully cohesive failure has been obtained for the case of CMLs subjected to different loading conditions. This approach creates a tough and compliant load transfer platform from the adhesive to the metal, which shields the composite core from failure.
- Finally, the use of adherends reinforced with toughened layers follows an approach which is similar to that of CMLs, as it is also able to provide fully cohesive failure. This method allows for the mitigation of the peel stress concentrations in the adhesive layer, virtually eliminating failure by delamination of the substrate.

## Data Availability

Not applicable.
